# When exome analysis is the key for your patient with cognitive decline: a case report

**DOI:** 10.1590/1980-5764-DN-2025-0384

**Published:** 2026-06-15

**Authors:** Antonio Avelino Mendes, Igor Fortunato da Silva, Daniel Santos Uchoa, Isabela Caldeira de Oliveira, Julian Leticia de Freitas, Maria Sheila Guimarães Rocha, Sonia Maria Dozzi Brucki

**Affiliations:** 1 Hospital Santa Marcelina, Departamento de Neurologia, São Paulo SP, Brazil.

**Keywords:** Leukoencephalopathies, Eukaryotic Initiation Factor-2B, Cognitive Dysfunction, Genetics., Leucoencefalopatias, Fator de Iniciação 2B em Eucariotos, Disfunção Cognitiva, Genética.

## Abstract

Vanishing white matter (VWM) disease is a leukodystrophy caused by mutations in *EIF2B1–5* genes, which impair cellular stress responses and protein synthesis regulation, leading to astrocytic dysfunction and white matter degeneration. While typically a pediatric condition, adult-onset cases are increasingly recognized. We report a 39-year-old Brazilian woman with a six-year history of progressive cognitive decline, gait impairment, and rapid deterioration following an infection. Neurological examination revealed spastic tetraparesis and severe cognitive impairment. Brain magnetic resonance imaging (MRI) showed diffuse white matter abnormalities with cystic degeneration. Whole-exome sequencing identified a homozygous *EIF2B3* c.260C>T (p.Ala87Val) variant, previously associated with a founder effect in Quebec but not reported in Brazil. This case highlights the phenotypic variability of VWM, stress-triggered exacerbations, and the importance of genetic testing in adult patients with cognitive decline. It also expands the genotypic diversity of VWM in Brazil and underscores the need for multidisciplinary care.

## RESUMO

A doença *vanishing white matter* (VWM) é uma leucodistrofia causada por mutações nos genes *EIF2B1-5*, que prejudicam as respostas ao estresse celular e a regulação da síntese proteica, levando à disfunção astrocitária e à degeneração da substância branca. Embora seja tipicamente uma condição pediátrica, casos de início na vida adulta vêm sendo cada vez mais reconhecidos. Relatamos o caso de uma mulher brasileira de 39 anos, com história de seis anos de declínio cognitivo progressivo, comprometimento da marcha e rápida deterioração após uma infecção. O exame neurológico revelou tetraparesia espástica e comprometimento cognitivo grave. A ressonância magnética cerebral evidenciou anormalidades difusas da substância branca, com degeneração cística. O sequenciamento completo do exoma identificou uma variante em homozigose em *EIF2B3* c.260C>T (p.Ala87Val), previamente associada a um efeito fundador no Quebec, mas não relatada no Brasil. Este caso destaca a variabilidade fenotípica da VWM, as exacerbações desencadeadas por estresse e a importância do teste genético em pacientes adultos com declínio cognitivo. Ele também amplia a diversidade genotípica da VWM no Brasil e ressalta a necessidade de cuidados multidisciplinares.

## INTRODUCTION

Vanishing white matter (VWM) disease, also termed childhood ataxia with central hypomyelination (CACH), is a progressive leukodystrophy caused by biallelic mutations in the *EIF2B1–5* genes, which encode subunits of the eukaryotic translation initiation factor 2B (eIF2B) complex^1^. This complex regulates cellular stress responses and protein synthesis. Its dysfunction leads to astrocytic failure and subsequent white matter degeneration^1,2^. Despite being a rare disease, *EIF2B5* mutations are a leading genetic cause among adult-onset leukoencephalopathies^3^.

Classically a pediatric disorder, VWM manifests with motor regression, ataxia, and spasticity, often triggered by stressors such as infection or trauma^2,4^. However, the disease can manifest at any age. Adult-onset cases are characterized by cognitive decline, psychiatric symptoms, and primary ovarian failure in female patients^2,4^. These patients differ significantly from pediatric forms, underscoring the importance of recognizing their specific features for accurate diagnosis and management^1^.

Here, we describe the first published case of adult-onset VWM in the Brazilian population due to an EIF2B3 variant and review the literature on its genetics, epidemiology, clinical manifestations, and diagnostic workup.

## CASE REPORT

A 39-year-old woman, with 14 years of schooling, was previously independent and employed as a saleswoman. She was admitted for evaluation of a six-year history of progressive cognitive decline and gait impairment. Initially, she presented with impoverished speech and slowed gait, including difficulty climbing stairs. Her symptoms progressed gradually over the following five years, during which she continued living alone but faced challenges securing employment, attributed to psychomotor slowness. One year before evaluation, her family reported a rapid deterioration in cognition and motor skills after a possible dengue virus infection. Her gait became markedly unstable, necessitating wheelchair use for most of the day, alongside significant trunk and limb incoordination. She also developed auditory/visual hallucinations and persecutory delusions.

**Figure 1:**
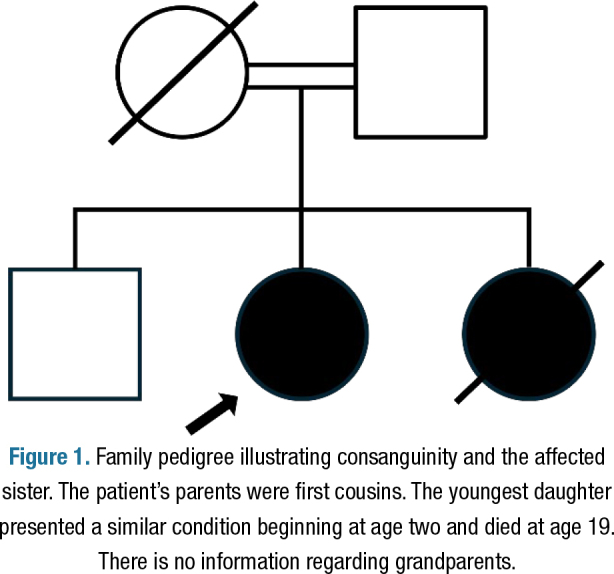
Family pedigree illustrating consanguinity and the affected sister. The patient’s parents were first cousins. The youngest daughter presented a similar condition beginning at age two and died at age 19. There is no information regarding grandparents.

Her medical history included recurrent migraines since adolescence (3–4 episodes weekly) and irregular menstrual cycles predating neurological symptoms. She denied comorbidities or toxic substance exposure. Family history revealed consanguineous parents (first cousins) and a sister with a similar condition, exhibiting symptom onset at age two, rapid progression, and death at 19 years old. An older brother remained asymptomatic (Figure 1).

Neurological examination disclosed: right hemifacial paresis on static and dynamic inspection. Global muscle strength grade IV (Medical Research Council scale) on counterposition testing. Hyperreflexia in all limbs, bilateral extensor plantar reflex, and a magnetic gait. Dystonic posture in the upper limbs. Dysmetria in both upper and lower extremities. Sensory examination was intact, with no abnormalities detected. Funduscopic examination revealed normal optic discs and retinal vessels. The cognitive assessment revealed a remarkable impairment (Table 1). She was dependent in instrumental activities of daily living but independent in basic activities.

**Table 1:** Performance on brief cognitive tests.

Test		Score/maximum score
Mini-Mental State Examination		13/30
Brief Cognitive Screening Battery	Naming	10/10
	Incidental memory	1/10
	Immediate memory	3/10
	Learning	5/10
	Delayed recall	9/10
	Semantic verbal fluency (animal)	3
	Clock drawing test	1/10
Addenbrooke’s Cognitive Examination-Revised	Attention and orientation	8/18
	Memory	3/26
	Fluency	0/14
	Language	15/26
	Visuospatial	6/16
	Total score	32/100

**Figure 2:**
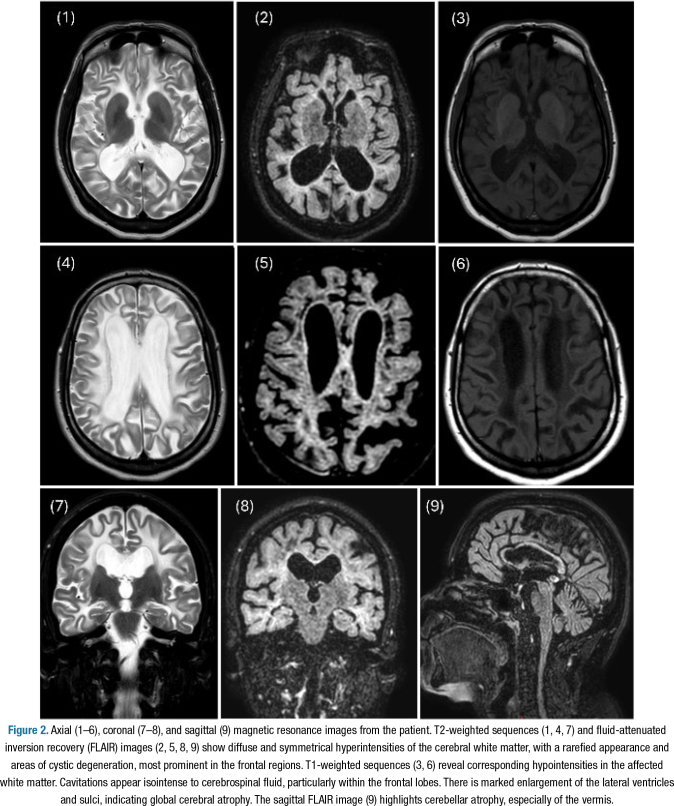
Axial (1–6), coronal (7–8), and sagittal (9) magnetic resonance images from the patient. T2-weighted sequences (1, 4, 7) and fluid-attenuated inversion recovery (FLAIR) images (2, 5, 8, 9) show diffuse and symmetrical hyperintensities of the cerebral white matter, with a rarefied appearance and areas of cystic degeneration, most prominent in the frontal regions. T1-weighted sequences (3, 6) reveal corresponding hypointensities in the affected white matter. Cavitations appear isointense to cerebrospinal fluid, particularly within the frontal lobes. There is marked enlargement of the lateral ventricles and sulci, indicating global cerebral atrophy. The sagittal FLAIR image (9) highlights cerebellar atrophy, especially of the vermis.

Laboratory investigations showed elevated follicle-stimulating hormone (FSH) and low estradiol levels (<5 ng/dL), consistent with primary ovarian failure. Serological, rheumatological, and metabolic panels (including calcium, copper, and iron studies) were unremarkable, as was cerebrospinal fluid analysis, ruling out inflammatory hypotheses. Brain MRI demonstrated diffuse, symmetric cerebral white matter abnormalities (T2 hyperintensity, T1 hypointensity) and frontal cystic lesions on fluid-attenuated inversion recovery (FLAIR) sequences (Figure 2), suggestive of leukodystrophy. There was no anomalous enhancement with gadolinium or calcifications in the susceptibility weighted imaging (SWI).

Due to the history of consanguineous parents with a sister presenting similar clinical manifestations, ovarian failure, and imaging showing rarefaction of the white matter with areas of cystic degeneration, VWM disease was suspected. Whole-exome sequencing identified a biallelic variant in EIF2B3, chr1:44978349G>A, corresponding to NM_020365.5:c.260C>T (p.Ala87Val) previously reported as pathogenic, confirming the diagnosis. It was not possible to perform Sanger sequencing nor to test her father or her brother.

After the initial hospitalization, the patient was referred to physical therapy, speech therapy, and endocrinology. We informed the family members about the need to avoid stressors such as trauma and infections. Therefore, they were advised to update their vaccinations, particularly against influenza and COVID-19, to avoid contact with individuals with upper respiratory infections, and to wear masks in public settings. However, a few months after the diagnosis was confirmed, the patient developed a urinary tract infection and experienced significant neurological deterioration, losing the ability to walk and developing bilateral tonic-clonic seizures.

## DISCUSSION

The patient presented a six-year history of insidious cognitive and motor deterioration, culminating in rapid decline after a febrile illness. A family history of a similarly progressive disorder in a younger sister supported a hereditary etiology. Brain MRI revealed marked atrophy of the cerebral white matter with diffuse T2-weighted hyperintensity, T1 hypointensity, and frontal cystic degeneration, consistent with VWM. Whole-exome sequencing presented a variant in the *EIF2B3* gene (c.260C>T, p.Ala87Val), which has multiple reports on ClinVar as a pathogenic variant^5^, establishing the diagnosis.

VWM is caused by biallelic mutations in any of the *EIF2B* genes. While *EIF2B5* mutations account for the most significant proportion of cases, *EIF2B3* variants are responsible for approximately 7.8%^2^. Variants are usually missense^1,2^ and the c.260C>T (p.Ala87Val) mutation in the *EIF2B3* gene was previously associated with a founder effect in the French-Canadian population of Quebec^6^. Our patient has family origins in the Northeast region of the country, and there is no European or Canadian ancestry that they are aware of.

Notably, pathogenic or likely pathogenic variants in *EIF2B3* in the Brazilian population before this case have not been published, and the A87V is absent in the national population database (ABraOM)^7^ — underscoring its novel identification in this demographic. The missense variant has a very rare frequency in international databases such as gnomAD (<0.05%), with no homozygotes reported^8^.

The mechanism of disease is loss of function of the eIF2B complex^1,2^. Despite being a genotype-phenotype correlation, intrafamilial variation can occur, particularly in those families that present adult-onset cases^2,4^. Additionally, asymptomatic individuals with biallelic *EIF2B* mutations have been identified through incidental MRI findings, highlighting the variable penetrance and expressivity of VWM^2^.

Adult-onset cases reported with A87V mutation have similar clinical findings to the case reported here, such as age of onset^6,9^, cognitive decline^6,9,10^, and ovarian failure (also termed ovarioleukodystrophy)^6,9,10^. Contrarily, there are few patients described with early-juvenile onset^6,11^ and other history data, e.g., hemiplegic migraines^6,10^. Some reports also present cases with compound heterozygosis^10,11^. The natural history study conducted by Hamilton et al. included three patients with the p.Ala87Val variant, and the age of onset ranged from two to 22 years old, with a duration of “lost walking without support” ranging from one to 11 years^4^.

Generally, juvenile (4–8 years) and adult-onset forms have predominantly cognitive decline and psychiatric symptoms, with motor deficits emerging later^2,4^. This cognitive impairment commonly includes deficits in executive functions (such as planning, organization, and abstract reasoning) and in memory (both short-term and working memory)^12,13,14^. Cognitive deterioration often occurs alongside other neurological symptoms like gait disturbances or psychiatric manifestations. Still, it can be the presenting symptom in adults, sometimes leading to initial misdiagnosis as primary progressive multiple sclerosis^9,12,14^. Dementia is a recognized outcome in adult-onset VWM, particularly as the disease advances^13^.

Regarding extracerebral involvement, Wei et al. reviewed 32 cases from the literature, and found that 72,7% had ovarian failure preceding the neurological symptoms^13^. Typical clinical findings include irregular menses, infertility, and amenorrhea^4^. The ovarioleukodystrophy phenotype results from rapid follicular atresia^1^. One patient reported having menometrorrhagia^13^.

Congenital and early-infantile forms (<1 year) present with severe encephalopathy, seizures, and rapid decline, often culminating in death within months^1,2^. Early juvenile-onset cases (2–4 years) typically present with ataxia, spasticity, and motor regression, while cognitive function remains relatively preserved initially^2,4^.

MRI remains the cornerstone of VWM diagnosis. It demonstrates bilateral, symmetrical involvement of the cerebral white matter, characterized by diffuse hypointensity on T1- and hyperintensity on T2-weighted images^2,15^. Early disease stages typically show non-cavitary white matter changes, while progressive cystic degeneration, manifesting as a radiating meshwork or dot-like pattern of cerebrospinal fluid-isointense cavitations, becomes prominent in advanced cases^15^. Cerebellar atrophy particularly affects the vermis^2^.

When faced with a white matter disease, it is crucial to consider the differential diagnoses. X-linked adrenoleukodystrophy is one of the most common genetic causes of leukodystrophy and typically shows a preferential parieto-occipital involvement, with lesions demonstrating contrast enhancement^15,16^. Krabbe disease also has a predilection for posterior regions, whereas metachromatic leukodystrophy more prominently involves frontal and periventricular areas, with sparing of the U-fibers — but those three diseases rarely present cystic degeneration^2,15,16^.

Mitochondrial leukoencephalopathies can exhibit more significant cystic degeneration than VWM, although accompanied by different clinical features and inheritance pattern^2^. Cerebral autosomal dominant arteriopathy with subcortical infarcts and leukoencephalopathy (CADASIL) has specific features that point towards its diagnosis, such as involvement of the temporal poles and external capsule, ischemic lacunae, and microhemorrhages^15^. It is also important to ponder acquired diseases such as multiple sclerosis in the differential diagnosis, but often the family history and symmetry of MRI findings point to a genetic disorder^2,15^.

Management of VWM remains supportive, with a focus on alleviating symptoms and avoiding stressors. The patient’s post-presumed dengue fever deterioration underscores the need for proactive measures to prevent infections, which can trigger catastrophic declines^4,12,13^. Multidisciplinary care, encompassing physical therapy, seizure management, and endocrine support for ovarian failure, is crucial for optimizing quality of life. Genetic counseling is critical, given the 25% recurrence risk in siblings^2^.

It is important to emphasize that limited access to comprehensive genetic testing often results in prolonged diagnostic odysseys for patients with adult-onset leukodystrophies. Delayed diagnosis may lead to misclassification as acquired demyelinating or neurodegenerative disorders, postponing appropriate genetic counseling, surveillance for systemic complications, and preventive strategies against stressors known to precipitate disease worsening^2,4^.

The Brazilian National Policy for Comprehensive Care for People with Rare Diseases, implemented in 2014, has fostered the expansion of services and the incorporation of genetic tests into public health strategies^17^. However, epidemiological data on rare diseases remain scarce, and many centers affiliated with the public Unified Health System (SUS) lack local access to comprehensive genetic testing. Additionally, genetic testing and specialized care are concentrated mainly in tertiary university hospitals located in major urban centers in the South and Southeast regions. At the same time, vast geographic areas continue to face limited availability of trained professionals and molecular diagnostic resources^18^.

This case expands the genotypic diversity of VWM in Brazil, reporting the first case of the *EIF2B3* c.260C>T (p.Ala87Val) variant in the population, previously absent from a national population database^7^. It reinforces the pivotal role of MRI and molecular analysis in adult patients presenting with cognitive decline and underscores the need for comprehensive, multidisciplinary care in managing VWM.

## Data Availability

The datasets generated and/or analyzed during the current study are not publicly available due to ethical restrictions, but are available from the corresponding author upon reasonable request.

## References

[1] Bugiani M, Vuong C, Breur M, van der Knaap MS (2018). Vanishing white matter: a leukodystrophy due to astrocytic dysfunction. Brain Pathol.

[2] van der Knaap MS, Fogli A, Boespflug-Tanguy O, Abbink TEM, Schiffmann R (2001). In: GeneReviews®.

[3] Lynch DS, Paiva ARB, Zhang WJ, Bugiardini E, Freua F, Lucato LT (2017). Clinical and genetic characterization of leukoencephalopathies in adults. Brain.

[4] Hamilton EMC, van der Lei HDW, Vermeulen G, Gerver JAM, Lourenço CM, Naidu S (2018). Natural history of vanishing white matter. Ann Neurol.

[5] Landrum MJ, Lee JM, Riley GR, Jang W, Rubinstein WS, Chirch DM (2014). ClinVar: public archive of relationships among sequence variation and human phenotype. Nucleic Acids Res.

[6] Robinson ME, Rossignol E, Brais B, Rouleau G, Arbour JF, Bernard G (2014). Vanishing white matter disease in French-Canadian patients from Quebec. Pediatr Neurol.

[7] Naslavsky MS, Scliar MO, Yamamoto GL, Wang JYT, Zverinova S, Karp T (2022). Whole-genome sequencing of 1,171 elderly admixed individuals from Brazil. Nat Commun.

[8] Karczewski KJ, Francioli LC, Tiao G, Cummings BB, Alföldi J, Wang Q (2020). The mutational constraint spectrum quantified from variation in 141,456 humans. Nature.

[9] Herwerth M, Schwäger BJ, Kreiser K, Hemmer B, Ilg R (2015). Adult-onset vanishing white matter disease as differential diagnosis of primary progressive multiple sclerosis: a case report. Mult Scler.

[10] La Piana R, Vanderver A, van der Knaap MS, Roux L, Tampieri D, Brais B (2012). Adult-onset vanishing white matter disease due to a novel EIF2B3 mutation. Arch Neurol.

[11] Wongkittichote P, Mar SS, McKinstry RC, Nguyen H (2022). Case report: a novel EIF2B3 pathogenic variant in central nervous system hypomyelination/vanishing white matter. Front Genet.

[12] Gui M, He M, Qin L (2024). Adult-onset leukoencephalopathy with vanishing white matter with compound heterozygous EIF2B3 gene variants. BMC Neurol.

[13] Kong F, Zheng H, Liu X, Li S, Wang J, Guo Z (2022). Association between late-onset leukoencephalopathy with vanishing white matter and compound heterozygous EIF2B5 gene mutations: a case report and literature review. Front Neurol.

[14] Wei C, Qin Q, Chen F, Zhou A, Wang F, Zuo X (2019). Adult-onset vanishing white matter disease with the EIF2B2 gene mutation presenting as menometrorrhagia. BMC Neurol.

[15] Resende LL, Paiva ARB, Kok F, Leite CC, Lucato LT (2019). Adult leukodystrophies: a step-by-step diagnostic approach. Radiographics.

[16] Thakkar RN, Patel D, Kioutchoukova IP, Al-Bahou R, Reddy P, Foster DT (2024). Leukodystrophy imaging: insights for diagnostic dilemmas. Med Sci (Basel).

[17] Brasil. Ministério da Saúde. Gabinete do Ministro. Portaria no 199, de 30 de janeiro de 2014 (2014). Institui a Política Nacional de Atenção Integral às Pessoas com Doenças Raras com Diretrizes para Atenção Integral às Pessoas com Doenças Raras no âmbito do Sistema Único de Saúde (SUS) e institui incentivos financeiros de custeio.

[18] Horovitz DDG, Félix TM, Ferraz VEF (2024). Medical Genetics in Brazil in the 21st Century: a thriving specialty and its incorporation in public health policies. Genes (Basel).

